# Utility of rapid prototyping in Complex DORV: does it alter management decisions?

**DOI:** 10.1186/1532-429X-18-S1-P175

**Published:** 2016-01-27

**Authors:** Puneet Bhatla, Sujata Chakravarti, Larry A Latson, Daniel K Sodickson, Ralph S Mosca, Nicole Wake

**Affiliations:** 1 Center for Advanced Imaging Innovation and Research (CAI2R) and Bernard and Irene Schwartz Center for Biomedical Imaging, Department of Radiology, New York University School of Medicine, New York, NY USA; 2 Department of Pediatrics, New York University School of Medicine, New York, NY USA; 3 Department of Cardiothoracic Surgery, New York University School of Medicine, New York, NY USA; 4 The Sackler Institute of Graduate Biomedical Sciences, New York University School of Medicine, New York, NY USA

## Background

Complex ventricular-arterial (VA) relationships in patients with double outlet right ventricle (DORV) make preoperative assessment of potential repair pathways challenging. The relationship of the ventricular septal defect (VSD) to one or both great arteries must be understood and this influences the choice of surgical procedure [1] In neonates and infants with DORV, Computed Tomography (CT) is often performed due to the ability to get high spatial resolution and ECG gated images [2], however it is possible to get the necessary information from Magnetic Resonance (MR) imaging with an added advantage of avoiding exposure to ionizing radiation. Both CT and MR allow image acquisition in three dimensions (3D) but traditional viewing of the anatomy using the multiplanar reformatting is actually done in two dimensions (2D). Volume rendering from either modality may also be performed, but typically only the external vascular anatomy is depicted. We hypothesized that it is possible to accurately define the intracardiac anatomy in infants with DORV using virtual and physical 3D printed (rapid prototyped) models created from either MR or CT and this can both aid in better defining potential VA pathways and may assist in surgical decision making.

## Methods

**Figure 1:**
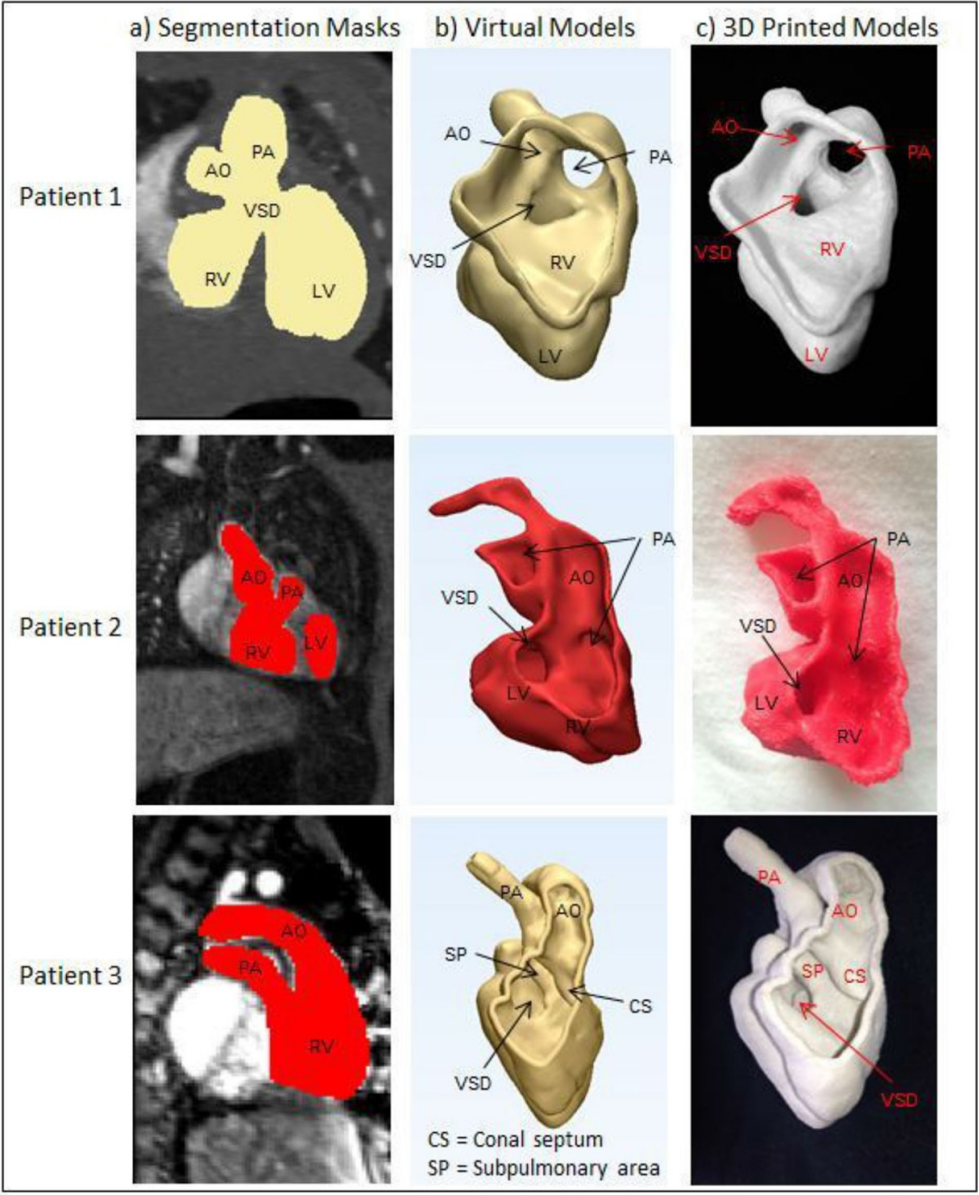
**a) segmentation masks, b) virtual models, and c) 3D printed models for all patients**.

Virtual and physical 3D models were generated for three patients with DORV. Non-ECG-gated 3D spoiled fast gradient echo sequence MR angiography was used for two patients. Retrospective ECG gated CT angiography images acquired in diastole were used in the third patient (to better define the coronary arteries given the suspicion of a single coronary artery by echocardiography). Blood pool segmentation (Figure 1a) was performed in all the three patients (Mimics, Materialise, Leuven, Belgium). A 2 mm shell was added to the blood pool and it was hollowed to create a patient specific heart replica (3-matic, Materialise, Leuven, Belgium). All virtual models were cut to best demonstrate the VA relationships and the models were printed.

## Results

The VSD and VA relationships were well visualized in all three patients using both the virtual and physical models (Figure 1b,c). The models helped the surgeons better understand the anatomy in all patients: in two patients the surgical plan was altered while the plan was confirmed in the third patient (Table 1).

**Table 1:** Patient Demographics and Surgical Plan

	Age	Diagnosis	Imaging Modality	Initial Surgical Plan	Additional Information Gained from Model	Final Surgical Intervention
Patient 1	1 week	DORV (S,D,D), Doubly committed VSD	CTA	VSD to Ao baffle	VSD-Ao baffle interferes with the RV to PA pathway	Yasui with RV to PA conduit
Patient 2	6 months	DORV (S,D,D), unclear VA relationship	MRA	VSD to PA baffle with arterial switch	Area of tunnel like sub PS is inferior to the LV-VSD-Ao pathway	BT shunt with future plan of LV to Ao baffle and RV-PA conduit
Patient 3	8 weeks	DORV (S,D,D), Subpulmonary VSD, pulmonary stenosis	MRA	Glenn surgery	Confirmed that potential attachment site of AV valve interferes with the VSD-Ao baffle	Glenn surgery

## Conclusions

Construction of 3D models in patients with DORV is feasible and allows for extensive examination and surgical planning. This may facilitate a focused and informed surgical procedure and improve the potential for successful outcome. For purposes of DORV, non-gated MRA is sufficient to delineate the VA relationships adequately for 3D printing and enhanced clinical decision-making. CT imaging should be reserved for only those patients where additional information like coronary artery anatomy is desired.

